# Treating *ARID1A* mutated cancers by harnessing synthetic lethality and DNA damage response

**DOI:** 10.1186/s12929-022-00856-5

**Published:** 2022-09-19

**Authors:** Jayaprakash Mandal, Prativa Mandal, Tian-Li Wang, Ie-Ming Shih

**Affiliations:** 1 BP Koirala Institute of Health Sciences, Dharan, Nepal; 2 Department of Gynecology and Obstetrics, Johns Hopkins University School of Medicine, Baltimore, USA; 3 Department of Pathology, Johns Hopkins University School of Medicine, Baltimore, MD USA

**Keywords:** ARID1A, Chromatin remodeling, Synthetic lethality, Cancer

## Abstract

Chromatin remodeling is an essential cellular process for organizing chromatin structure into either open or close configuration at specific chromatin locations by orchestrating and modifying histone complexes. This task is responsible for fundamental cell physiology including transcription, DNA replication, methylation, and damage repair. Aberrations in this activity have emerged as epigenomic mechanisms in cancer development that increase tumor clonal fitness and adaptability amidst various selection pressures. Inactivating mutations in *AT-rich interaction domain 1A* (*ARID1A*), a gene encoding a large nuclear protein member belonging to the SWI/SNF chromatin remodeling complex, result in its loss of expression. *ARID1A* is the most commonly mutated chromatin remodeler gene, exhibiting the highest mutation frequency in endometrium-related uterine and ovarian carcinomas. As a tumor suppressor gene, *ARID1A* is essential for regulating cell cycle, facilitating DNA damage repair, and controlling expression of genes that are essential for maintaining cellular differentiation and homeostasis in non-transformed cells. Thus, ARID1A deficiency due to somatic mutations propels tumor progression and dissemination. The recent success of PARP inhibitors in treating homologous recombination DNA repair-deficient tumors has engendered keen interest in developing synthetic lethality-based therapeutic strategies for *ARID1A*-mutated neoplasms. In this review, we summarize recent advances in understanding the biology of ARID1A in cancer development, with special emphasis on its roles in DNA damage repair. We also discuss strategies to harness synthetic lethal mechanisms for future therapeutics against *ARID1A*-mutated cancers.

## Background

Targeting the DNA damage response (DDR) for cancer therapy has gained significant attention in recent years. This promise reflects the fact that many malignant neoplasms are defective in their DNA damage repair machineries, most commonly a result of molecular genetic alterations, either somatic or inherited, in genes critically involved in various repair pathways. The recent success of PARP inhibitors in treating homologous recombination-deficient ovarian high-grade serous carcinomas champions such endeavors and encourages basic scientists and oncologists to explore novel anti-cancer strategies on a basis of synthetic lethality [1, 2]. Those efforts aimed to further impair the specific DNA damage repair pathway and overload DNA damage beyond the repair capacity in cancer cells with a compromised repair system. In particular, alongside the traditional concept of targeting DDR enzymes such as PARP1, WEE1, and ATR, harnessing the epigenomic changes in cancer cells represents another exciting direction as DNA damage repair largely depends on chromatin configuration [3]. The purpose of this review is to discuss how inactivating mutations in *AT-rich interaction domain 1A (ARID1A)*, a component of the chromatin remodeling SWI/SNF complex, affect DNA damage repair in tumor cells. First, we briefly review the diverse key cellular functions of ARID1A. Next, we highlight the therapeutic potential by targeting the ARID1A synthetic lethal phenotype with inhibitors of PARP, ATR, or other agents in *ARID1A-*mutated tumors. We will also provide our insights into the promises and challenges in developing therapeutic strategies to treat cancers with *ARID1A* mutations.

## ARID1A and chromatin remodeling mechanism

**Fig. 1:**
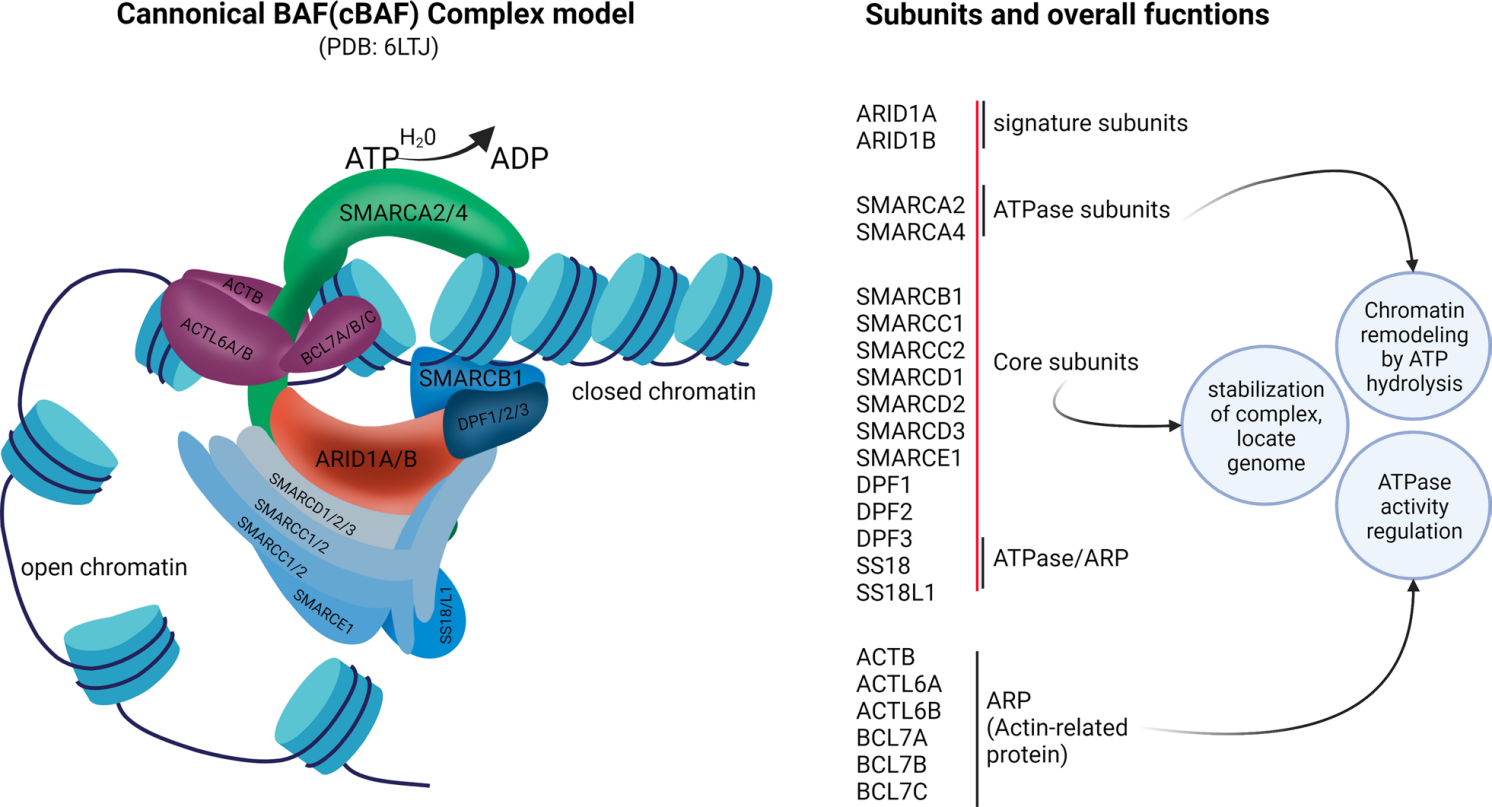
Subunits of ARID1A-containing BAF complex and their overall function. Model of canonical BAF (cBAF) complex illustrating the remodeling of closed chromatin to open chromatin structure driven by ATP hydrolysis. The mutually exclusive core subunits ARID1A/ARID1AB (ARID1A/B) are depicted in shaded orange. On the right panel, cBAF-subunits, their class/module, and overall function in chromatin remodeling are shown [16, 17]. Illustration created using Biorender.com

The SWItch/Sucrose Non-fermentable (SWI/SNF) was originally identified in *saccharomyces cerevisiae* in 1984 [4, 5]. The human form of SWI/SNF complex is known as BRG1/BRM associated factor complex (BAF complex) or mammalian SWI/SNF (mSWI/SNF) complex. The subunits of mSWI/SNF complex, which are encoded by 29 genes, are assembled into three distinct complexes: canonical BAF (cBAF), polybromo-associated BAF (PBAF), and non-canonical BAF (ncBAF) [6]. These subunits bind to the distal enhancer regions, promoter regions, and CCCTC-binding factor (CTCF)-binding sites at many genomic loci, facilitating DNA accessibility to various nuclear proteins and their complexes so they can perform their functions. A BAF complex uses an ATPase core to hydrolyze ATP, generating energy to relax heterochromatin (condensed form) into euchromatin (relaxed form) by nucleosome unwrapping, mobilization, and exchanging [7] (Fig. 1). BAFs also bind to several other nuclear proteins, and these mechanisms collectively enhance the accessibility of nuclear factors for transcriptional activation, DNA duplication, and DNA repair. Conversely, the polycomb repressor complex restores the heterochromatin state after BAF complex disengagement [8].

AT-rich interaction domain 1A (ARID1A) is one of the chromatin remodeling proteins belonging to the SWI/SNF complex. The cBAF complex contains several subunits within which ARID1A and ARID1B are mutually exclusive (Fig. 1). ARID1A is also known as Brahma-related associated factor 250a (BAF250a), SWI/SNF-related matrix-associated actin-dependent regulators of chromatin factor 1 (SMARCF1), B120, C1orf4, Osa1, or p270 [9, 10]. The gene encoding ARID1A is located on chromosome 1p36.11. ARID1A, a large (250 kDa) nucleocytoplasmic protein, is actively imported from cytoplasm to the nuclear compartment. Nuclear ARID1A is degraded by the ubiquitin–proteasome system but its dynamic import–export across nuclear membrane favors its nuclear accumulation in normal cells as evidenced by its universal expression in nuclei from normal cells [11]. Thus, ARID1A has been reported to directly interact with DNA via its ARID domain using a consensus motif [12].

In humans, germline *ARID1A* mutation has been thought to be a genetic culprit in the Coffin-Siris syndrome, a neurodevelopmental disorder characterized by congenital malformation and developmental delay (OMIM: #614607) [13]. In rodent models, *Arid1a* transcripts and protein levels are highly increased during all stages of mouse embryonic development and its ablation leads to the loss of pluripotency, self-renewal of mouse embryonic stem (ES) cells, and severe developmental arrest [14, 15].

## ARID1A as a tumor suppressor

The discovery of *ARID1A* as a tumor suppressor gene was made more than a decade ago by two sequencing-based studies. Both studies analyzed whole exomes and global mRNAs in rare subtypes of epithelial ovarian cancer, clear cell carcinoma, and endometrioid carcinoma and identified a high frequency of  inactivating mutations occurring diffusely in *ARID1A* with resultant loss of protein expression, a characteristic of a tumor suppressor. The mutations lead to truncated proteins that are rapidly degraded, and thus, ARID1A is functionally inactivated. Subsequently, inactivating *ARID1A* mutations have been detected in a wide variety of human cancers, highlighting the epigenomic roles of ARID1A in cancer development [18, 19].

Loss of ARID1A expression can also be a result of *ARID1A* promoter hypermethylation [20], and it is likely that tumors use sequence mutations in both alleles, or promoter hypermethylation together with mutations in different alleles, to drive complete loss of ARID1A proteins, following the classical “two-hit” theory characterizing tumor suppressor genes. The resultant loss of ARID1A expression impairs the interaction of ARID1A with other SWI/SNF subunits in the nucleus [11]. Occasionally, in-frame insertions and deletions affecting the nuclear transporting signal are also detected in cancer tissues, and those mutants also result in loss of its nuclear expression [11].

Approximately ~ 6% of human cancers harbor inactivating mutations in *ARID1A*. Some of the cancer types showing the highest incidence of *ARID1A* inactivating mutations and loss of expression are clear cell ovarian cancer (~ 50%), endometrial cancers (~ 37%), gastric cancers (20–30%), bladder cancers (~ 20%), hepatocellular cancers (~ 14%), melanomas (~ 12%), colon cancers (~ 9%), and lung cancers (~ 8%) [21–23]. *ARID1A* mutations have also been studied for their potential role as biomarkers for predicting clinical outcomes in cancer patients [24].

In vitro cell models and genetically engineered mouse models have provided cogent evidence to support the functional role of ARID1A in tumor suppression [12, 25–29]. Those studies have also elucidated multiple mechanisms involved. Of most relevance, ARID1A regulates expression of proteins participating in cell cycle control and DNA repair after DNA damage and replication stress. Loss of ARID1A in colon and ovarian cancer cells results in epigenetic reprogramming at enhancer regions and reduction of the open chromatin mark, H3K27ac, subsequently leading to loss of transcription of nearest genes. ARID1A drives the BAF-complex to enhancer regions and helps maintain chromatin in the relaxed configuration [25, 30]. Other mechanisms that are affected due to ARID1A loss are defective control of cell cycle progression, DNA damage checkpoint, regulation of p53 downstream targets (CDKN1A and SMAD3), and regulation of telomerase activity [31, 32]. ARID1A is also involved in decatenation of newly replicated sister chromatids [33]. Defects in the decatenation checkpoint is responsible for aneuploidy and genomic instability [34]. In the following sections, we briefly describe the roles of ARID1A in transcriptional regulation and maintenance of DNA integrity in the context of DNA damage and replication stress.

## Transcriptional regulation by ARID1A

ARID1A-mediated transcriptional regulation is mainly attributed to its chromatin accessibility role. ARID1A can increase or decrease chromosome accessibility histone marks at the promoter regions of genes and regulate their expression. ARID1A-mediated chromatin remodeling regulates transcription positively as well as negatively [35]. Generally, acetylation of histones (histone mark: H3K27ac) keeps the chromatin open, permitting DNA transcription to take place, whereas methylation of histones (histone mark: H3K9me3) keeps the chromatin closed, preventing transcription. For instance, ARID1A depletion decreases the open histone mark (H3K27ac) at the enhancer region and transcriptionally activates a number of cancer related genes including *PIK3IP1* [36], *SLC7A11* [37], *CDKN1A*, *TGF-β* receptor [12], and *SMAD3* [38] among several others.

On the other hand, ARID1A depletion promotes expression of the deubiquitinating enzyme USP9X. Mechanistically, ARID1A recruits HDAC1 to the promoter of USP9X where it downregulates its expression by removing the acetyl group from histone. Conversely, depletion of ARID1A causes an increase in the acetylation level H3K9 and H3K27 at the USP9X promoter, thereby increasing its expression [39]. Similarly, cells lacking ARID1A show increased transcription of *HDAC6*, *AURKA*, and *TERT* [31] associated with increased histone acetylation. ARID1A is seen to control global transcription by controlling RNA polymerase II (RNAPII) dynamics. ARID1A depletion represses RNAPII, which causes transcriptional dysregulation of several active genes. Although restoration of a mutually exclusive partner, ARID1B, rescues transcription of some of these genes, targets of p53 and estrogen receptor genes are not rescued [40].

## Role of ARID1A in DNA damage response

**Fig. 2:**
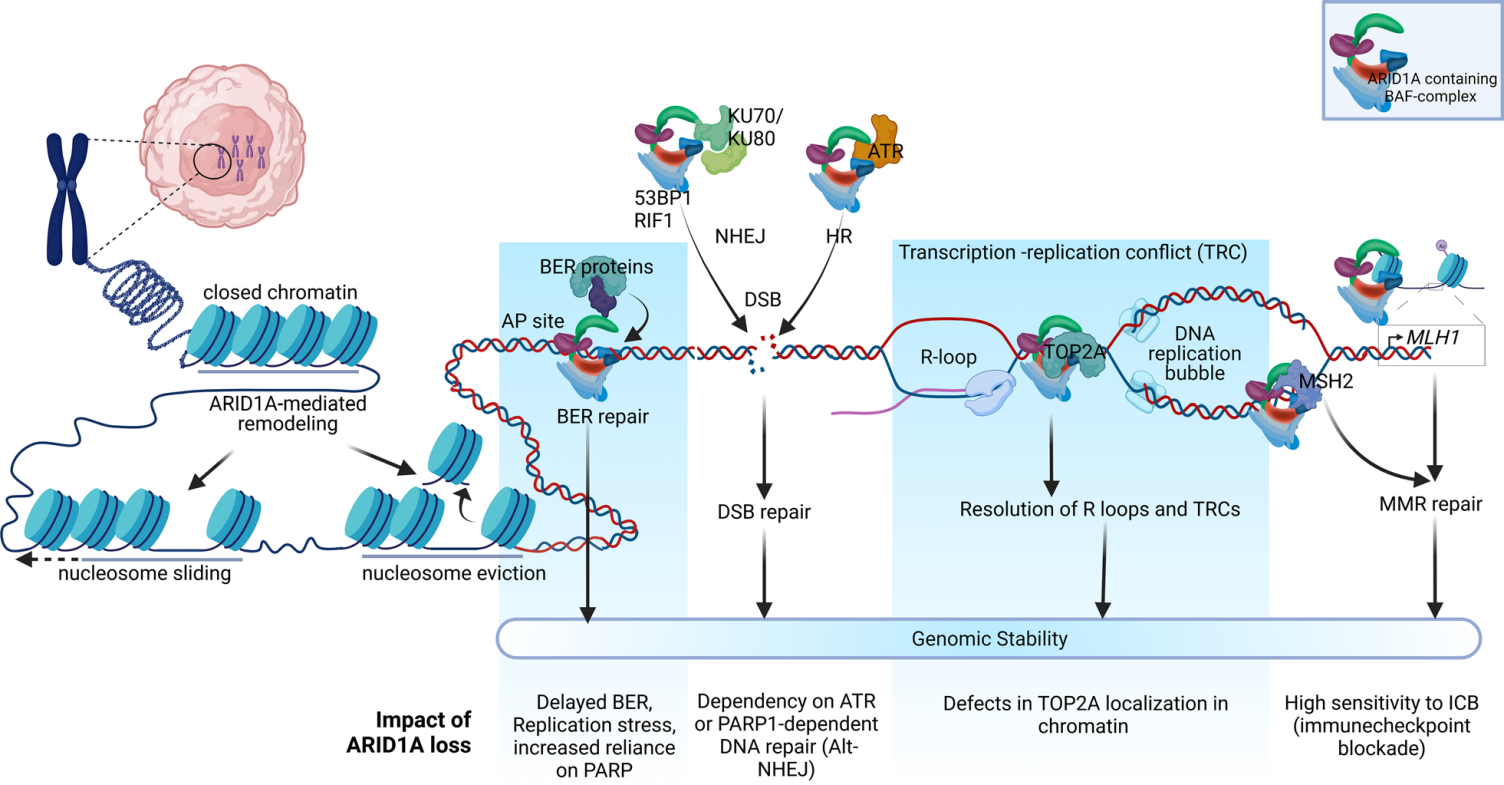
Chromatin remodeling and role of ARID1A in maintaining genome integrity. Chromatin remodeling (nucleoside sliding and nucleosome eviction) by ARID1A-containing BAF complex is shown at left (see Fig. 1). Chromatin remodeling is required to open the chromatin structure to ensure easy accessibility of DNA repair proteins of various DNA repair pathways as shown on the right. ARID1A is directly involved in repairing DNA damage using DNA repair mechanisms such as BER, MMR, NHEJ, Alt-NHEJ, and HR and in resolving TRC and aberrant R-loops that give rise to replication stress. *ARID1A* AT-Rich Interaction Domain 1A, *AP site* apurinic/apyrimidinic site, *BER* base excision repair, *PARP* poly adenosine diphosphate-ribose polymerase, *53BP1* tumor suppressor p53-binding protein 1, *RIF1* Replication Timing Regulatory Factor 1, *NHEJ* non-homologous end joining, *HR* homologous recombination, *Alt-NHEJ* alternate non-homologous end joining, *DSB* double-strands break, *ATR* ataxia telangiectasia and Rad3-related protein, *TRC* transcription-replication conflict, *TOP2A* DNA Topoisomerase II Alpha, *MSH2* MutS homolog 2, *MMR* mismatch repair, *MLH1* MutL homolog 1, *ICB* immune checkpoint blockade. Illustration created using Biorender.com

In recent years, the emerging roles of ARID1A in the DNA damage response (DDR) have been established (Fig. 2). These new data not only furnish the means for understanding the fundamental biology of DDR but also suggest mechanisms for synthetic lethality when *ARID1A* is inactivated. DNA damage and replication stress initiate DDR through activities of two signaling proteins that detect these aberrant events: ATM (ataxia-telangiectasia mutated) and ATR (ATM and Rad3-Related). ATM is generally activated by a double-strand DNA break (DSB) whereas ATR is activated by a single-stand break (SSB), DNA replication stress [41], and DNA-end resection (which generates a single-strand DNA region during DSB repair). In S-phase, ATR is responsible for replication initiation, replisome stability, and replication fork restart, whereas in G2-phase it prevents premature mitotic advance. Inhibition of ATR interferes with the cell cycle and genomic stability, thus decreasing cell survival [42]. DNA DSBs are repaired by several major pathways in mammalian cells: non-homologous end joining (NHEJ), and homologous recombination (HR) repair pathways. In case of defective NHEJ or HR, alternative NHEJ provides a backup mechanism involving Poly (ADP-ribose) polymerase 1 (PARP1).

ARID1A-containing SWI/SNF complexes also allow DNA repair proteins to effectively access DNA damage sites [43]. Inactivation of SWI/SNF complex by deleting or knockdown of ARID1A compromises DNA DSB repair, increases sensitivity to DNA damaging agents, and impairs γ-H2AX induction [44]. Specifically, suppression of ARID1A reduces NHEJ by decreasing the recruitment of NHEJ factors such as KU70/KU80 and the ATPase subunit of SWI/SNF complex to the DSB sites [45]. ARID1A knockout cells are unable to mount efficient NHEJ repair after irradiation, but largely spare PARP-mediated alt-NHEJ and DSB repair pathways. This is because 53BP1 and RIF1, key NHEJ factors, delay to arrive at the DSB sites due to ARID1A deficiency. As expected, a combination of low-dose irradiation and PARP inhibitor shows a synergistic cytotoxicity in ARID1A-deficient tumor cells in both in vitro and in vivo models [43].

ARID1A is also involved in repairing damaged DNA through homologous recombination (HR) repair. Mechanistically, ARID1A is recruited to DNA DSB sites through interacting with the upstream kinase, ATR. ARID1A also helps recruit the ATPase subunit of the SWI/SNF complex to DNA damage sites. Loss of ARID1A also impairs the G2/M DNA damage checkpoint. Overall, ARID1A facilitates DSB end resection and also helps maintain checkpoint signaling [46]. Thus, ARID1A protects the genome by interacting with the machinery of different DNA repair mechanisms.

## ARID1A, DNA mismatch repair, and immune checkpoint

Alterations that lead to loss of expression of any genes such as *MSH2*, *MSH6*, *MLH1*, and *PMS2* or their combination in the mismatch repair (MMR) pathway contributes to DNA MMR deficiency, especially in endometrial and colorectal carcinomas. When the MMR pathway is defective, tumors show a high microsatellite instability (MSI-H) phenotype, producing up to thousands of somatic mutations in the genome, including many that encode neo-antigens which potentially trigger immune responses. *ARID1A* mutations have been found enriched in MSI-H neoplasms including endometrial carcinomas [47–49], ovarian carcinomas [50], gastric adenocarcinomas [51, 52], colon cancers [53, 54], and prostate cancers [53]. It remains uncertain if ARID1A is mechanistically  involved in the mismatch repair process or if the *ARID1A* mutations are simply the result of the unstable hyper-mutated phenotype. There is some evidence supporting the former. In endometrial and colorectal cancer, ARID1A loss is associated with *MLH1* silencing resulting from promoter hypermethylation [49, 55]. As well, ARID1A has been shown to promote MMR by recruiting MSH2 to chromatin during DNA replication [56]. These findings imply that ARID1A plays a causal role in MMR.

Many ARID1A-mutated endometrial and colorectal carcinomas harbor the MSI-H phenotype. This finding is of significant clinical interest as the MSI-H phenotype provides a biomarker relevant to immunotherapy based on anti-PD1/PD-L1 drugs [54]. ARID1A transcriptionally regulates PD-L1 expression. *CD274*, which encodes PD-L1, is transcriptionally repressed by ARID1A [57]. As a result, loss of ARID1A predicts increased PD-L1 expression [58] via activating AKT signaling [59, 60]. These data suggest that ARID1A loss may help cancer cells, especially those with MMR deficiency, escape from immune checkpoint surveillance through upregulation of PD-L1.

## Role of ARID1A in replication stress response

Replication stress characterizes cancer and is associated with incessant and uncontrolled cell replication [61]. Replication stress arises when replication forks are hindered by DNA abnormalities such as DNA lesions [62], R-loops, and secondary DNA structures. The replication stress response represents a deviation from a normal DNA replication program in rapidly dividing cancer cells with poor quality control during replication. In response to various DNA replication problems, activation of ATR kinase and its downstream effector kinase CHK1 is responsible for the replication stress response [63, 64]. ATR phosphorylates checkpoint kinases 1 and 2 (CHK1 and CHK2) to stall the replication cycle when single-stranded DNA breaks mount, giving cells time to repair their DNA. There are several sources of replication stress as discussed below.

### R-loops

R-loops are three-stranded hybrid nucleic acid structures formed during a normal transcription process (Fig. 2). Physiologically, R-loops are involved in transcription initiation-termination, Ig class switch recombination, and telomerase elongation [65]. When the homeostasis of an R-loop is altered, the progression of replication is stalled by the phenomenon of transcription-replication conflict. During this conflict, machineries of DNA replication and transcription collide [66] leading to replication stress. If the stress is not effectively resolved in a timely manner, persistence of R-loops can become the source of replication stress and subsequent genomic instability [67, 68]. The presence of a persistent R-loop causes activation of the ATR pathway through an as yet unknown mechanism [69, 70]. Aberrant R-loops that fail to be resolved interfere with DNA damage repair and transcription [71] (reviewed in [68]). Recently, loss of ARID1A has been reported to lead to replication stress and R-loop formation [72]. ARID1A loss generates replication stress through DNA topoisomerase (TOP2A) mis-localization in the genome. By modulating DNA topology, TOP2A is involved in chromosome condensation, separation of chromatids, DNA replication, and transcription [73, 74]. Thus, ATR activation is critical for mitigating R-loop aberrations, and the ATR pathway is responsible for protecting the genome by suppressing transcription-replication conflicts, promoting replication-fork recovery, and arresting cells at G2-M phase for further repair. These new data suggest that ATR inhibition may preferentially kill ARID1A-deficient tumors.

### DNA catenation

Another source of replication stress is DNA catenation, an intermediate product of DNA replication. It is formed when two replication forks collide and DNA topoisomerase II removes these structures (known as decatenation) [75]. Mechanistically, TOP2A-mediated DNA decatenation requires BAF complex subunits such as BRG1 (SMARC4) and BAF250A (ARID1A) to bind to chromatin. In the absence of ARID1A, TOP2A is unable to complete this process before mitosis, and as a result, complex chromosomal structures can arise during DNA replication. Moreover, ARID1A loss leads to DNA decatenation defects in tumor cells, which is thought to be due to improper localization of TOP2A [33, 73]. TOP2A helps resolve topological issues brought on by chromosomal metabolism, such as catenated DNA and transcription-replication conflicts. It appears that the failure of TOP2A to properly localize in concert with delayed cell cycle progression in ARID1A deficient cells would result in an increased requirement for ATR function, suggesting a therapeutic potential of ATR inhibitor.

### Apurinic/apyrimidinic site

Apurinic/apyrimidinic sites (AP sites), also known as abasic sites, are DNA repair intermediates that act as roadblocks to replication fork progression [61]. These lesions are generally repaired, but replicative DNA polymerases using the Translesion Synthesis (TLS) pathway can bypass these lesions if they are not resolved in a timely fashion [76]. Alkylating drugs like temozolomide and mitomycin-C methylate DNA bases and those methylated bases are repaired by both base excision repair (BER) and nucleotide excision repair (NER) [77]. Recently, we have observed that that ARID1A-deficient cancer cells challenged with temozolomide and PARP inhibitor exhibited significant replication stress, replication fork instability, and DNA damage due to impaired BER arising from the ARID1A deficiency, leading to cell death (Fig. 2). We showed that ARID1A-deficient cancer cells developed a high susceptibility to PARP inhibitors in the presence of TMZ and link BER and TLS functionally. Loss of ARID1A delayed arrival of repair proteins at the AP sites compromising BER. Compromised BER and TLS may exacerbate replication fork stalling in ARID1A-deficient cells, resulting in fork collapse and cell death. Moreover, our preclinical study warrants further clinical testing for the FDA-approved alkylating agent TMZ and PARPi combination, which exhibits synergistic benefits in the ARID1A-deficient tumors (manuscript submitted).

## Therapeutic targeting of DDR pathway-synthetic lethality and combination

**Fig. 3:**
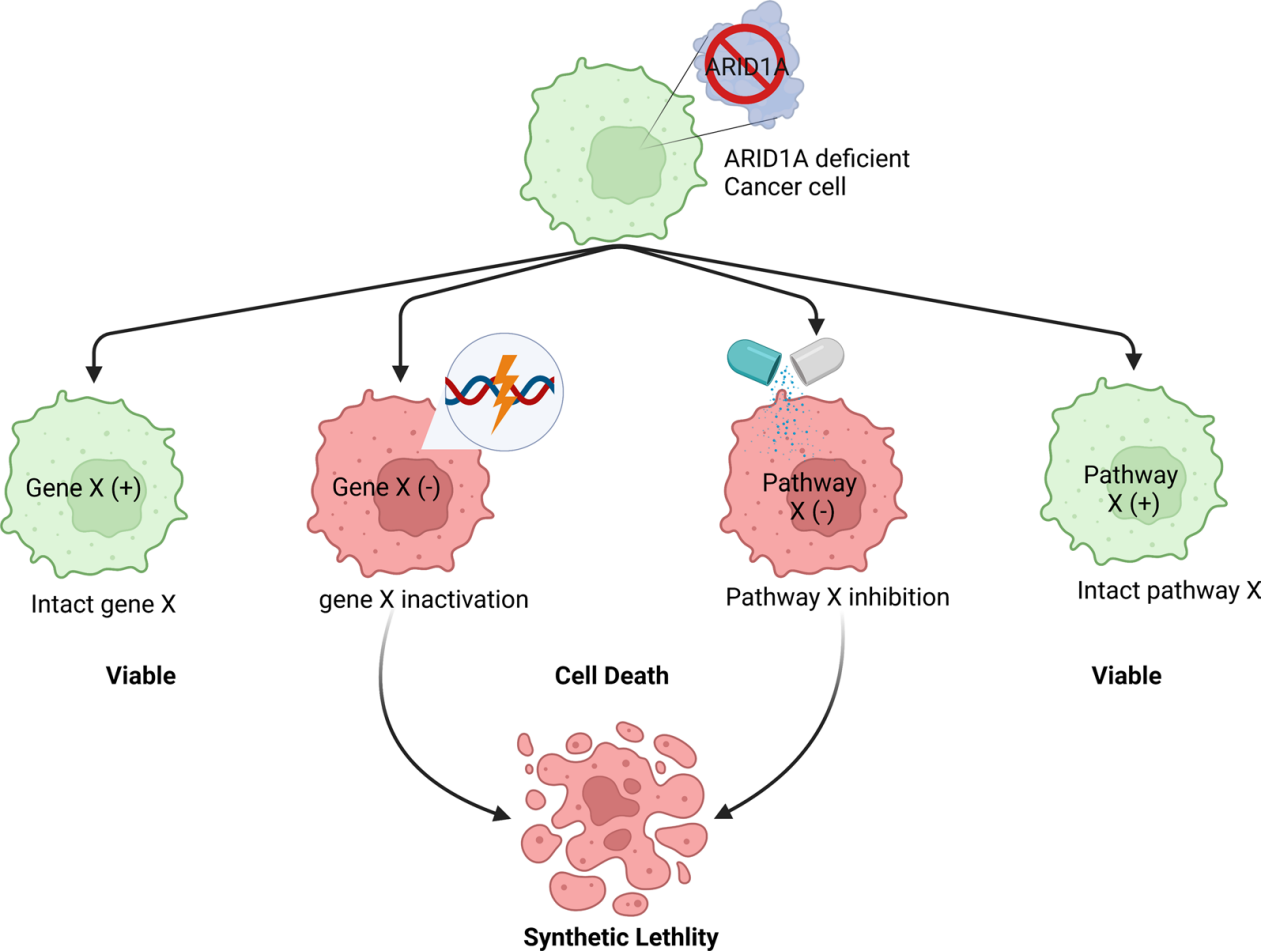
Schematic representation of ARID1A-based synthetic lethality. Synthetic lethal interaction or synthetic lethality between two genes occurs when their simultaneous inactivation results in cell death. In this illustration, two synthetic lethal partners are ARID1A and a hypothetical gene ‘X’ or a pathway that gene ‘X’ regulates. In a malignant cell having ARID1A loss (top green cell), viability is dependent upon its essential gene ‘X’. Loss of gene ‘X’ either by inactivating mutation or using drug/inhibitor results in cell death. Illustration created using Biorender.com

Synthetic lethality is a phenomenon where the simultaneous loss of function of both genes causes cell death, whereas the inactivation of any one of two genes independently has no impact on cell survival. The idea of synthetic lethality in cancer has been expanded to include pairs of genes, where the inactivation of one gene through deletion or mutation and the pharmaceutical inhibition of the other results in the death of cancer cells, whereas normal cells are spared they do not have the fixed genetic alteration [78]. Since its discovery as a tumor suppressor, ARID1A has entered center stage as an agent for synthetic lethal drug screening. Synthetic lethal interactions provide a key avenue for development of cancer therapies. In anti-cancer therapy, by principle, inhibiting the functional products of such lethal gene partners should kill the cancer cells [79, 80] (Fig. 3). A landmark achievement based on synthetic lethality in cancer was the discovery of poly (ADP-ribose) polymerase (PARP) inhibitor in *BRCA1/2* mutated ovarian cancer [1, 2]. In the following, we will discuss the promise in ARID1A synthetic lethal phenotypes using PARP inhibitor, ATR inhibitor, and other emerging agents.

## Targeting ATR and PARP

**Fig. 4:**
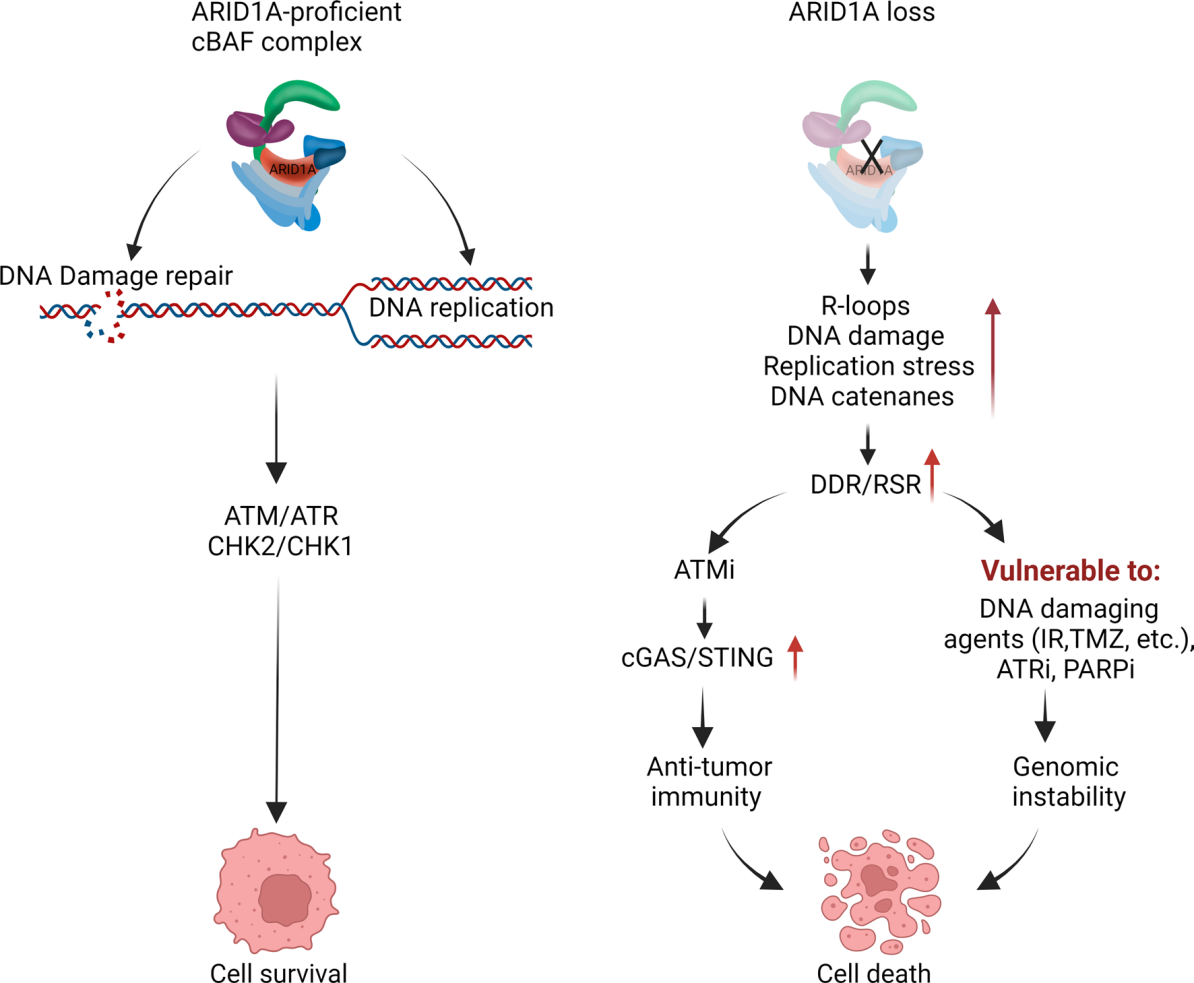
Overview of targeting DNA damage response pathway in the context of ARID1A-deficiency (see text for description). Illustration created using Biorender.com

It has become clear that loss of ARID1A impairs genome stability after DNA damage. Since genomic stability is essential for normal cells to survive, several repair pathways have evolved to protect the genome. It is not surprising that upon ARID1A loss, other pathways will compensate for its loss of function. Thus, targeting those complementary pathways holds promise in developing new cancer therapies in ARID1A deficient neoplasms. The role of ATR, PARP1, and TOP2A in maintaining genome integrity is pivotal as discussed in the previous section. To reiterate, after ARID1A loss, ATR signaling is activated as a result of replication stress due to transcription-replication conflicts, aberrant R-loop formation, defective decatenation process, and DNA damage. Thus, inhibition of ATR can theoretically eliminate ARID1A-deficient cancer cells. In fact, ATR inhibitors have been reported to act in a synthetic lethal way in ARID1A-deficient cancer [73]. ATR inhibition has been shown to disrupt BRCA1-independent loading of RAD51 at DSBs causing stalled forks and to be effective in overcoming resistance to PARP inhibitor in cancer cells exhibiting BRCAness [81]. As several ATR inhibitors have entered into clinical studies, targeting ATR holds promise to treat ARID1A mutated cancers. In fact, a combination of ATR inhibitor (ceralasertib) and PARP inhibitor (olaparib) has been proposed as a model to overcome PARP inhibitor resistance (NCT02576444, Table 1) in patients with relapsed or refractory malignancies exhibiting DDR changes. ATR inhibitor may elicit ATM activation to compensate for its inactivation (Fig. 4). This feature provides a rationale for targeting both stress kinases (ATM/ATR) in ARID1A-deficient cancers. Recently, it was reported that ARID1A-deficient tumors exhibited high expression of Chk2. Loss of ARID1A altered E3-ligase RNF8-mediated degradation of Chk2. Since Chk2 is a downstream kinase of ATM, inhibition of the ATM/Chk2 axis in ARID1A deficient cells led to replication stress, increased tumor-infiltrating lymphocytes, and a STING-mediated innate immune response resulting in longer patient survival [82].

**Table 1:** Selected clinical trials involving ARID1A in DDR and beyond; https://clinicaltrials.gov (accessed: 07.18.2022)

Target group	Clinical trial ID	Study title	Drug (target)	Disease	Study phase	Status
Targeting DDR, ICB and combination	NCT02278250	First in human study of M4344 in participants with advanced solid tumors	M4344 (ATRi)	Advanced solid tumors	1	Completed
	NCT03718091	M6620 (VX-970) in selected solid tumors	M6620 (ATRi)	Solid tumor Leiomyosarcoma Osteosarcoma	2	Completed
	NCT04042831	Olaparib in treating patients with metastatic biliary tract cancer with aberrant DNA repair gene mutations	Olaparib (PARPi)	Biliary tract cancer	2	Recruiting
	NCT03207347	A trial of niraparib in BAP1 and other DNA damage response (DDR) deficient neoplasms (UF-STO-ETI-001)	Niraparib (PARPi)	Mesothelioma Uveal melanoma Renal cell Carcinoma Cholangiocarcinoma	2	Active, not recruiting
	NCT04716686	Niraparib monotherapy as maintain and recurrent treatment of endometrial serous carcinoma	Niraparib (PARPi)	Endometrial carcinoma Serous carcinoma	2	Recruiting
	NCT04065269/ATARI	Atr inhibitor in combination with olaparib in gynecological cancers with arid1a loss or no loss	AZD6738 (ATRi) Olaparib (PARPi)	Gynecological cancers	2	Recruiting
	NCT03682289	Phase II trial of AZD6738 alone and in combination with olaparib	AZD6738 (ATRi) Olaparib (PARPi)	Advanced cancers	2	Recruiting
	NCT02576444/OLAPCO	Olaparib combinations	AZD6738 (ATRi) AZD2281 (PARPi) AZD5363 (AKTi) AZD1775 (WEE1i)	Cancer	2	Active, not recruiting
	NCT04633902	Phase II study of olaparib and pembrolizumab in advanced melanoma with homologous recombination (HR) mutation	Olaparib (PARPi) Pembrolizumab (PD-1i)	Metastatic melanoma	2	Recruiting
	NCT04953104	Nivolumab for the treatment of patients with metastatic urothelial cancer with ARID1A mutation and stratify response based on CXCL13 expression	Nivolumab (PD-1i)	Urothelial cancer or solid tumors	2	Not yet recruiting
	NCT04957615	Nivolumab for the treatment of metastatic or unresectable solid tumors with ARID1A mutation and CXCL13 expression	Nivolumab (PD-1i)	Metastatic malignant solid neoplasm Unresectable solid neoplasm	2	Not yet recruiting
Targeting epigenetics, ICB and combination	NCT05154994/RESOLVE	Tremelimumab, durvalumab, and belinostat for the treatment of ARID1A mutated metastatic or unresectable, locally advanced urothelial carcinoma	Belinostat (HDACi) Durvalumab (PD-L1i) Tremelimumab (CTLA-4i)	Urothelial carcinoma	1	Recruiting
	NCT04493619	PLX2853 as a single agent in advanced gynecological malignancies and in combination with carboplatin in platinum-resistant epithelial ovarian cancer	PLX2853 (BETi) Carboplatin	Gynecologic neoplasms	1, 2	Active, not recruiting
	NCT03297424	A Study of PLX2853 in Advanced Malignancies	PLX2853 (BETi)	Advanced malignancies	1, 2	Completed
	NCT05023655	Phase II study of tazemetostat in solid tumors harboring an ARID1A mutation	Tazemetostat (EZH2i)	Solid tumors	2	Recruiting
	NCT03348631	Tazemetostat in treating patients with recurrent ovarian or endometrial cancer	Tazemetostat (EZH2i)	Recurrent ovarian or endometrial cancer	2	Suspended
	NCT04104776	A study of CPI-0209 in patients with advanced solid tumors and lymphomas	CPI-0209 (EZH2i)	Advanced solid tumor and lymphomas	1, 2	Recruiting
Targeting kinases	NCT02059265	Dasatinib in treating patients with recurrent or persistent ovarian, fallopian tube, endometrial or peritoneal cancer	Dasatinib (Multikinase inhibitor)	Recurrent gynecologic cancer	2	Active, not recruiting
	NCT01914510	A study of ENMD-2076 in ovarian clear cell cancers	ENMD-2076 (Multikinase inhibitor)	Ovarian clear cell carcinoma	2	Completed

*i* inhibitor, *ATR* ataxia telangiectasia and Rad3-related inhibitor, *PARP* poly adenosine diphosphate-ribose polymerase, *PD-1* programmed cell death protein 1, *PD-L1* programmed death ligand-1, *CTLA-4* cytotoxic T-lymphocyte-associated protein 4, *HDAC* histone deacetylase, *BET* bromodomain and extra-terminal motif, *EZH2* enhancer of zeste homolog 2

In addition to ATR inhibition [73], TOP2A and PARP1 can also be targeted in ARID1A-deficient tumors as a combination therapy. These two molecules (ARID1A and ATR) are synthetic lethal partners [83] and increase the sensitivity to PARP inhibitors [46]. However, a known clinical challenge seen in PARP inhibitor is its acquired resistance that is best characterized by a BRCA reversion mutation that partially or fully restores its tumor suppressor function. Another challenge is the hematological toxicity associated with certain clinically used PARP inhibitors. Alongside PARP enzymatic inactivation, first-generation PARP inhibitors trap PARP1 and PARP2 at the DNA damage sites. Both repair proteins are required to activate the DDR pathway, and because of the trapping, cells cannot efficiently repair damaged DNA, leading to cytotoxicity not only for cancer cells but also for normal cells. The subsequent development of a selective PARP1 inhibitor showed a low level of toxicity and a high therapeutic index (AZD5305 phase I/II PETRA trial).

## Emerging non-DDR synthetic lethal partners

**Fig. 5:**
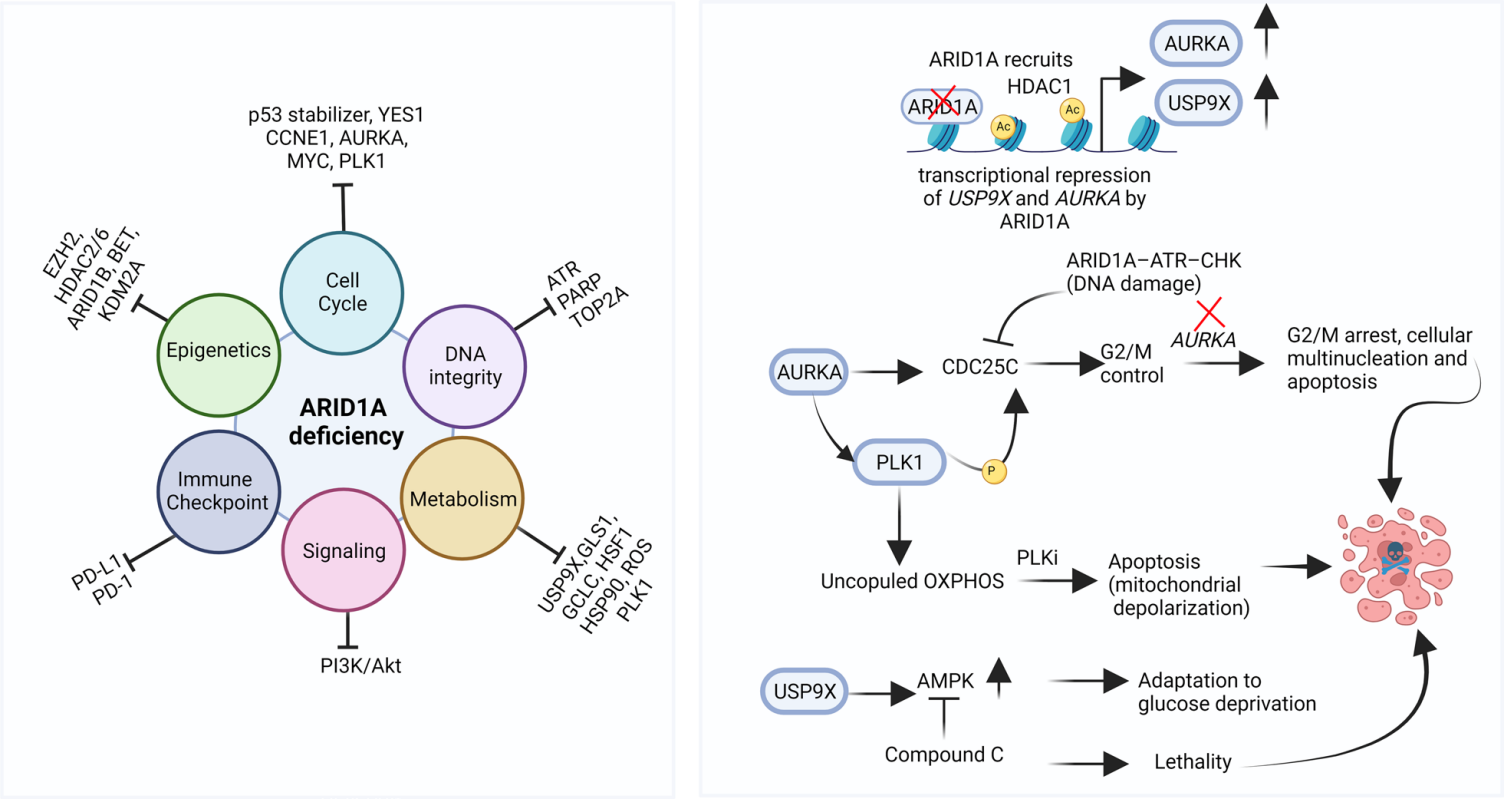
Synthetic lethal partners of ARID1A deficiency. The left panel illustrates various synthetic lethal targets of ARID1A deficiency belonging to diverse cellular functions such as regulating cell cycle, DNA protection, cellular metabolism, signaling pathways, epigenetic functions, and immune checkpoint regulation. The right panel illustrates mechanisms of synthetic lethality of new targets such as PLK1 and USP9X. The deubiquitinating enzyme USP9X is transcriptionally repressed by ARID1A, whose loss upregulates USP9X. USP9X upregulates AMPK (AMP-activated protein kinase) signaling for cellular survival under glucose deprivation. Targeting AMPK with dorsomorphin (Compound C) kills the cancer cells. Similarly, inhibition of PLK1 (Polo Like Kinase 1) induces apoptosis due to uncoupled Oxidative phosphorylation (OXPHOS). AURKA (Aurora kinase A) phosphorylates CDC25C through PLK1. CDC25C checks G2/M checkpoint. Loss of AURKA results in G2/M arrest. The DNA damage response also checks CDC25C [102]. Illustration created using Biorender.com

In addition to PARP and ATR, there are several existing (reviewed in: [83–88]) and emerging non-DDR synthetic lethal partners, targeting of which may lead to increased cytotoxicity in ARID1A-deficient tumors (Fig. 5).

Polo-like kinase 1 (PLK1) belongs to the serine-threonine kinase family. PLK1 plays a critical role in advancement of the cell cycle to mitosis by promoting mitotic spindle assembly, cell division, DNA-damage response, and maintenance of genomic stability. Overexpression of PLK1 has been reported to enhance proliferation rate and enhance tumorigenicity. Conversely, its inhibition causes reduction of proliferation and apoptosis in cancer cells but not in normal cells, suggestive of a potential role as a cancer therapeutic agent [89]. However, clinical progress using PLK1 inhibitor has been limited likely due to a lack of biomarkers capable of identifying patients whose tumors are vulnerable to this inhibitor. Recently, the role of PLK1 in ARID1A-deficient cancer cells has been reported. Cells lacking ARID1A are highly sensitive to PLK1 inhibition [90]. Interestingly, the sensitivity is related to mitochondrial metabolism rather than to the known role of PLK1 in cell division. *ARID1A* knockout cells exhibit altered mitochondrial biogenesis showing a higher number of globular mitochondria, increased oxidative phosphorylation, and increased oxygen consumption without increased ATP production. Interestingly, PLK1 is localized at mitochondria, and this may explain why PLK1 inhibition is associated with exaggerated mitochondrial oxygen consumption and membrane depolarization, leading to apoptosis. To further explore the therapeutic potential of targeting PLK1 in the context of ARID1A deficiency, PLK1 inhibition may be combined with chemotherapeutic agents and PARP inhibitor. To this end, PLK1 is reported to increase the temozolomide sensitivity in glioma stem cells [91]. Recently, the combination of temozolomide and PLK1 inhibitor has shown synergistic cytotoxicity in glioma cells in vivo [92].

USP9X (ubiquitin-specific peptidase 9 X-linked) is a deubiquitinase which regulates the protein levels of its substrates through proteasomal degradation. USP9X is involved in cancer initiation and development [93, 94], and intriguingly, its role in cancer has both pro-oncogenic [95] and tumor suppressor functions, likely depending on tissue context [96, 97]. High expression of USP9X confers resistance to gemcitabine and cisplatin in pancreatic cancer [98] and breast cancer [99], respectively. USP9X is also involved in DNA damage repair and DNA replication processes [100]. Loss of USP9X has been shown to cause accumulation of the double-strand break marker, γ-H2AX. This is likely because USP9X regulates the mRNA expression levels of BRCA1 and RAD51. Therefore, loss of USP9X decreases the BRCA1 and RAD51 mRNA levels, compromising their HR repair capacity. As expected, depletion of USP9X causes inefficient DSB repair and is synthetic lethal to PARP (poly (ADP-ribose) polymerase) inhibition [101].

Recently, the role of USP9X in ARID1A-deficient hepatocellular carcinoma was reported to involve a synthetic lethal interaction between ARID1A deficiency and inactivation of adenosine 5′-monophosphate-activated protein kinase (AMPK) [39]. Thus, a novel ARID1A-HDAC1-USP9X-AMPK axis in cancer is proposed. In non-transformed cells, ARID1A recruits HDAC1 which deacetylates H3K9 and H3K27 to epigenetically silence USP9X, and further downregulates its downstream substrate, AMPK. In a glucose-deprived condition, ARID1A-proficient cells undergo cell death following proteasomal degradation of protein kinase AMP-activated catalytic subunit a2 (PRKAA2) due to its role in silencing USP9X. However, in ARID1A-deficient cells, USP9X is upregulated and blocks proteasomal degradation by deubiquitinating PRKAA2 (K364 residue) conferring cell survival. Thus, targeting this AMPK pathway leads to cell death preferentially in ARID1A-deficient cancer cells. Further studies are warranted to validate this synthetic lethal effect in different types of human cancer.

## Prognostic potential of ARID1A

Synthetic lethality-based targeting vulnerabilities of ARID1A deficiency exploits the high mutational burden in many malignancies. Thus, ARID1A deficiency has the potential as a biomarker for precision medicine in various cancers. Loss of *ARID1A*, a tumor suppressor gene, has been shown to have significant prognostic value in several malignancies, gastric cancer [103–105], lung cancer [106], hepatocellular carcinoma [107–109], breast cancer [110], osteosarcoma [111], clear cell renal carcinoma [112, 113], and small intestinal carcinoma [114]. ARID1A loss has shown adverse (tumor progression, poor prognosis, lower survival, chemotherapy resistance, early recurrence), beneficial (enhance patient survival) or no effect on progression-free survival and overall survival of cancer patients (for review [115]). In gynecologic cancers, loss of ARID1A is also regarded as an independent prognostic biomarker. For instance, reduced ARID1A expression has been correlated with the FIGO (International Federation of Gynecology and Obstetrics) stage, shorter progression-free survival in ovarian clear cell carcinoma [116, 117], endometrium-related cancers, and cervical cancer [118]. Conversely, several studies reported having no difference between ARID1A-negative and ARID1A-positive staining groups in terms of clinical stage, grade, histopathologic features, and overall survival in various gynecologic malignancies [119–123]. There could be several factors involved for such ambiguous prognostic role of ARID1A within the same cancer type. Those factors include limited sample size, lack of common choice of anti-ARID1A antibodies (for IHC) or concomitant mutations like *PIK3CA*, *TP53*, *EZH2*, *KRAS* as in ARID1A-deficient gynecological cancers that impact the prognosis in these cancers. Moreover, it is rare for a single gene to serve as a significant prognostic marker. Instead, a group of markers may serve better for this purpose. For instance, ARID1A, p53, and β-Catenin in combination may have a better prognostic potential in ovarian clear cell and endometroid subtypes [124].

## Concluding remarks and perspectives

*ARID1A* has emerged as one of the major tumor suppressor genes in many types of human cancer, especially those arising from the endometrial epithelium. Somatic mutations of *ARID1A* have been shown to alter the chromatin landscape and to affect several fundamental cellular functions including transcription, DNA hypermethylation, DNA synthesis, and DNA damage response. In recent years, *ARID1A* mutation has been explored for synthetic lethal targeting, and certain epigenetic drugs and inhibitors in the DNA damage repair have shown promise for treating *ARID1A*-mutated tumors. Immune checkpoint blocking is also currently being evaluated for efficacy in controlling *ARID1A-*mutated cancers. Despite the promise, several challenges need to be overcome before *ARID1A*-based precision oncology becomes a reality.

Foremost, the functional difference between the SWI/SNF complex in the presence and absence of ARID1A remains to be fully determined. Because of the mutually exclusive nature of subunits ARID1A and ARID1B, when ARID1A is missing, ARID1B can substitute for ARID1A in SWI/SNF complexes in maintaining DNA accessibility to various nuclear proteins. The knowledge whether ARID1A-containing complex and ARID1B-containing complex show different specificity to bind to DNA loci or the subunit alter the chromatin remodeling activity is important to understand the pathogenesis in tumor cells losing ARID1A, and help discover the synthetic lethal strategies.

Second, identification and validation of biomarkers to predict treatment outcomes using synthetic lethal drugs like EZH2 inhibitor and PARP inhibitor is important for development of effective targeted therapies. Intuitively, *ARID1A* mutation or loss of expression can be an ideal tissue biomarker, but clinical validation is required to affirm this conclusion. It is uncertain if monoallelic inactivation (mutation, ch1p36 deletion, or epigenetic silencing) share some phenotypes as bi-allelic inactivation. Other biomarkers involved in various DNA damage repair pathways can also be explored for outcome correlations in ongoing and future clinical trials.

Third, despite the well-described clinical benefits of targeted cancer therapy, drug resistance to monotherapy is inevitable due to the expansion of tumor clones that are refractory to the targeted therapeutic agent under an ever-changing tumor microenvironment. Combinational therapy provides a possible solution for this significant clinical problem but increased adverse effects can become more apparent. A better dosing and scheduling strategy in a well-defined clinical setting is warranted to deliver effective therapy to *ARID1A*-mutated malignancies.

## Data Availability

Not applicable.

## Abbreviations

ARID1A: AT-Rich Interaction Domain 1A
AP site: Apurinic/apyrimidinic site
BER: Base excision repair
PARP: Poly adenosine diphosphate-ribose polymerase
53BP1: Tumor suppressor p53-binding protein 1
RIF1: Replication Timing Regulatory Factor 1
NHEJ: Non-homologous end joining
HR: Homologous recombination
Alt-NHEJ: Alternate non-homologous end joining
DSB: Double-strand break
ATR: Ataxia telangiectasia and Rad3-related protein
TRC: Transcription-replication conflict
TOP2A: DNA Topoisomerase II Alpha
MSH2: MutS homolog 2
MMR: Mismatch repair
MLH1: MutL homolog 1
ICB: Immune checkpoint blockade
PD-1: Programmed cell death protein 1
PD-L1: Programmed death ligand-1
CTLA-4: Cytotoxic T-lymphocyte-associated protein 4
HDAC: Histone deacetylase
BET: Bromodomain and Extra-Terminal motif
EZH2: Enhancer of zeste homolog 2
